# Homologs of SCAR/WAVE complex components are required for epidermal cell morphogenesis in rice

**DOI:** 10.1093/jxb/erw214

**Published:** 2016-06-01

**Authors:** Wenqi Zhou, Yuchuan Wang, Zhongliang Wu, Liang Luo, Ping Liu, Longfeng Yan, Suiwen Hou

**Affiliations:** Ministry of Education Key Laboratory of Cell Activities and Stress Adaptations, School of Life Sciences, Lanzhou University, Lanzhou 730000, China

**Keywords:** Epidermal cell, F-actin, lobe, morphogenesis, rice, SCAR/WAVE.

## Abstract

Filamentous actins (F-actins) play a vital role in epidermal cell morphogenesis. However, a limited number of studies have examined actin-dependent leaf epidermal cell morphogenesis events in rice. In this study, two recessive mutants were isolated: *less pronounced lobe epidermal cell2-1* (*lpl2-1*) and *lpl3-1*, whose leaf and stem epidermis developed a smooth surface, with fewer serrated pavement cell (PC) lobes, and decreased papillae. The *lpl2-1* also exhibited irregular stomata patterns, reduced plant height, and short panicles and roots. Molecular genetic studies demonstrated that *LPL2* and *LPL3* encode the PIROGI/Specifically Rac1-associated protein 1 (PIR/SRA1)-like and NCK-associated protein 1 (NAP1)-like proteins, respectively, two components of the suppressor of cAMP receptor/Wiskott-Aldrich syndrome protein-family verprolin-homologous protein (SCAR/WAVE) regulatory complex involved in actin nucleation and function. Epidermal cells exhibited abnormal arrangement of F-actins in both *lpl2* and *lpl3* expanding leaves. Moreover, the distorted trichomes of Arabidopsis *pir* could be partially restored by an overexpression of *LPL2*. A yeast two-hybrid assay revealed that LPL2 can directly interact with LPL3 *in vitro*. Collectively, the results indicate that LPL2 and LPL3 are two functionally conserved homologs of the SCAR/WAVE complex components, and that they play an important role in controlling epidermal cell morphogenesis in rice by organising F-actin.

## Highlights

This study revealed that two novel proteins in rice – LPL2 and LPL3 – homologous to the SCAR/WAVE subunits PIR/SRA1 and NAP1, respectively, control epidermal cell morphogenesis through actin reorganization.

## Introduction

The shape of plant cells, as determined by cell wall expansion, is critical for organ and tissue development and morphogenesis during plant growth (Smith, 2003; Mathur, 2004; Smith and Oppenheimer, 2005; Yanagisawa *et al.*, 2015). As a paradigm system, the epidermis (including leaf, stem, and root epidermis) is thought to be a common feature of almost all multicellular land plants that perform multi-faceted functions. It acts as a barrier, perceives external stimuli, transmits signals, secretes enzymes, and absorbs nutrients (Guimil and Dunand, 2006, 2007; Martin and Glover, 2007). In the model dicot plant Arabidopsis, the leaf epidermal cells are mainly composed of pavement cells (PCs), stomata, and trichomes (Guimil and Dunand, 2006, 2007). The development of each cell type represents an accessible model system for studying cell patterns and morphogenesis (Fu *et al.*, 2005; Bergmann and Sack, 2007; Qian *et al.*, 2009; Chen *et al.*, 2015; Yanagisawa *et al.*, 2015). Leaf epidermal PCs have an interlocking jigsaw puzzle shape, and their role is to protect plants via functions such as maintaining temperature and resisting foreign invasion (Glover, 2000; Qian *et al.*, 2009). Stomata and trichomes are morphologically specialized epidermal cells in plants. Stomata regulate the exchange of oxygen and carbon dioxide and control water transpiration between plants and the atmosphere (Glover, 2000; Bergmann and Sack, 2007; Peterson *et al.*, 2010). Leaf trichomes are responsible for protecting the growing organs against insect attack (Mauricio, 1998).

Rice (*Oryza sativa* L.) is a model monocotyledon plant because of its small, well-mapped genome size, shared single-nucleotide polymorphisms, and improved genetic transformation technology (Toki, 1997; Matsumoto *et al.*, 2005; Toki *et al.*, 2006). Furthermore, it is one of the most important food crops for more than half of the global population. And considering the vital role of the epidermis to plant success, studies focusing on epidermal cell morphogenesis in rice are needed. Thus far, only limited numbers of genes that regulate epidermis development have been described in rice. For instance, *Dwarf and Gladius Leaf 1* regulates the alignment and elongation of rice leaf epidermal cells (Komorisono *et al.*, 2005). *OsSPCH*, *OsMUTE*, and *OsFAMA* are involved in stomata cell and pattern development (Liu *et al.*, 2009; Peterson *et al.*, 2010). *Glabrous Rice 1* and *TUTOU1* (*TUT1*) mediate leaf trichome development and morphology (Li *et al.*, 2012; Bai *et al.*, 2015). *Wilted Dwarf and Lethal 1* and *Early Senescence 1* (*ES1*) affect epidermal cell differentiation. Knockout of these two genes leads to higher stomatal density and rapid water loss in rice (Park *et al.*, 2010; Rao *et al.*, 2015). Therefore, the plant epidermis plays an essential role in the growth process and physiological responses to biotic and abiotic stress in rice.

The cytoskeleton is extremely important for epidermal cell morphogenesis, and it is primarily regulated by cortical microtubule (MT) and cortical ﬁlamentous actin (F-actin) polymerization and stabilization in plants (Mathur, 2004; Fu *et al.*, 2005; Smith and Oppenheimer, 2005; Panteris *et al.*, 2006, 2007). Several studies on Arabidopsis have shown that the morphostructure of epidermal PCs with interdigitated lobes is controlled by MTs and/or F-actins in the leaf epidermis (Fu *et al.*, 2005; Armour *et al.*, 2015). In particular, the distorted trichomes and/or less bulged lobes of the epidermis are mainly controlled by F-actin rearrangement in Arabidopsis (Qiu *et al.*, 2002; Szymanski, 2005; Zhang *et al.*, 2008; Yanagisawa *et al.*, 2015). In a similar mechanism for monocotyledons, maize (*Zea mays*) *BRICK1* (*BRK1*), *BRK2*, and *BRK3* genes are involved in epidermal cell morphogenesis and cell division (Frank and Smith, 2002; Frank *et al.*, 2003, 2004; Panteris and Galatis, 2005; Panteris *et al.*, 2006; Facette *et al.*, 2015). These studies in maize highlighted the primary role of F-actins in leaf PC lobe formation among graminaceous plants.

F-actin polymerization during epidermal cell morphogenesis has been well documented in distorted trichomes in Arabidopsis, especially with regard to the suppressor of cAMP receptor/Wiskott-Aldrich syndrome protein-family verprolin-homologous protein (SCAR/WAVE) regulatory complex, and the actin-related protein 2/3 (Arp2/3) complex signaling pathway (Mathur, 2003; Basu *et al.*, 2004, 2005; Deeks *et al.*, 2004; El-Assal *et al.*, 2004; Li *et al.*, 2004; Zimmermann *et al.*, 2004; Szymanski, 2005; Djakovic *et al.*, 2006). In Arabidopsis, the SCAR/WAVE complex was shown to include five highly evolutionary conserved subunits: PIROGI/Specifically Rac1-associated protein (PIR/SRA1), NCK-associated protein (NAP1/NAP125), BRICK1/HSPC300, ABI, and SCAR (Basu *et al.*, 2004, 2005; Deeks *et al.*, 2004; El-Assal *et al.*, 2004; Saedler *et al.*, 2004; Zimmermann *et al.*, 2004; Zhang *et al.*, 2005; Djakovic *et al.*, 2006; Le *et al.*, 2006; Mendoza, 2013). The Arp2/3 complex consists of seven elements (ARP2, ARP3, ARPC1, ARPC2, ARPC3, ARPC4, and ARPC5) (Higgs and Pollard, 2001; Pollard, 2007). The Arp2/3 complex forms a nucleation site to begin a new actin filament branch and is vital for F-actin polymerization in plant cells (Frank *et al.*, 2004; Yanagisawa *et al.*, 2013). During F-actin polymerization, Arp2/3 is activated by the SCAR/WAVE protein via its conserved verprolin and coﬁlin homology and acidic (VCA) domain (Frank *et al.*, 2004; Uhrig *et al.*, 2007; Zhang *et al.*, 2008; Yanagisawa *et al.*, 2013). The SCAR/WAVE complex is stimulated by the active Rho-family GTPase of plants (Rac/Rop-GTPases) along with the subunit PIR/SRA1 (Basu *et al.*, 2004, 2005; Yanagisawa *et al.*, 2013). However, few genes have been found to control F-actin polymerization; they affect leaf epidermis morphogenesis in rice, except *TUT1*/*OsSCAR1*, which encodes the same SCAR-like protein. This protein plays a significant role in activating the Arp2/3 complex to regulate actin polymerization (Bai *et al.*, 2015; Rao *et al.*, 2015). In this study, two genes, *LPL2* and *LPL3*, encoding the PIR/SRA1-like protein and the NAP1-like protein, respectively, both of which are subunits of the SCAR/WAVE complex in rice, were investigated. They play an essential role in local F-actin rearrangement associated with lobe outgrowth in rice leaves.

## Materials and methods

### Plant materials

Rice (*Oryza sativa* ssp. *japonica* cv. Zhonghua 11, ZH11) and T-DNA insertion library seedlings with ZH11 ecotype background were cultivated in the greenhouse of Lanzhou University (Gansu, China), with 12-h light period/day, 60%–80% relative humidity, and day/night temperature of 32/22 °C. *lpl2-1* was screened and back-crossed into ZH11 three times prior to use. Two T-DNA insertion mutant lines *lpl2-2* (RMD_04Z11HY54) and *lpl2-3* (RMD_04Z11CR30) were ordered from the Rice Mutant Database, China (http://rmd.ncpgr.cn/introduction.cgi?nickname). The location of T-DNA insertions in *lpl2-2* was further verified by PCR using *LPL2* ﬂanking primers (LPL2-2FP-F and LPL2-2FP-R) and primer PFRB4-RB. We verified T-DNA of *lpl2-3* using primers LPL2-3FP-F, LPL2-3FP-R and NTLB5-LB. *lpl3-1* (ACNB06) was ordered from CIRAD, France (http://orygenesdb.cirad.fr/index.html), and its T-DNA insertions were verified by PCR using primers LPL3-FP-F/-R and LPL3 T-DNA speciﬁc primer (LPL3-SP). The *pir* T-DNA insertion mutant SALK_106757 (Alonso *et al.*, 2003) was ordered from the Arabidopsis Biological Resource Center (ABRC), and its T-DNA was verified by PCR using PIR-FP/SP primers. All PCR primers are listed in Supplementary Table S1 at *JXB* online.

### Dental resin impression and stomatal density

The dental resin impression method was used to screen mutants *lpl2-1*, *lpl2-2*, *lpl2-3* and *lpl3-1*. Fully expanded ~20-day-old rice leaves were used to impress the abaxial side, and the detailed impression procedures and stomatal density statistics (average number of stomata per mm^2^ of leaf) were performed using methods from our previous report (Luo *et al.*, 2012).

### Scanning electron microscopy (SEM)

The juvenile leaf and flag leaf blades were used for SEM analysis. All images were taken using a HITACHI S-3400N microscope with an acceleration voltage of 10kV. The impressions from the same tender leaf blade showed the consecutive developmental processes of epidermal cells, and scanning was performed on the abaxial surfaces in juvenile and mature leaves.

### Resin slice analysis

The epoxy resin semi-thin sections were taken from the same parts of the leaves by light microscopy according to the reference manual (Technovit 7 100 resin).

### Map-based cloning

The F2 population progenies of *lpl2-1* (*Oryza sativa* ssp. *japonica*) and 9311 (*Oryza sativa* ssp. *indica*) hybrids were selected using dental resin impressions for mapping. The published RM-series rice simple sequence repeat (SSR) markers (http://www.gramene.org/) were used to map the mutant gene. The locus was roughly mapped between RM4108 and RM4352 on the long arm of chromosome 3 by the primary location. Subsequently, the locus was fine mapped onto the ~98-kb region between two markers RM14374 and RM14380 using 880 *lpl2-1* homozygote. The candidate gene *LPL2* was identiﬁed by sequence analysis of all genes on the region.

### Plasmid construction and plant transformation

The full-length open reading frame (ORF) of *LPL2* was ampliﬁed using primers OE-LPL2-F and OE-LPL2-R with the KpnI and SpeI restriction enzyme sites, respectively (Supplementary Table S1), with reverse-transcribed cDNA as a template. Then the *LPL2* ORF was subcloned into an overexpression (OE) binary vector POX driven by maize ubiquitin promoter (from Liu Yaoguang laboratory) to obtain *OE-LPL2*, which was introduced into *lpl2-1* mutant embryonic calli by *Agrobacterium tumefaciens*-mediated transformation (Toki *et al.*, 2006). Plasmid *35S-LPL2-GFP* was constructed using the same method mentioned above based on the pCAMBIA-1300-GFP vector, and then was transformed into *pir* mutant plants (Clough and Bent, 1998). Phenotypic and genetic analyses were performed mainly in the T2 generation. All PCR primers are listed in Supplementary Table S1.

### Quantitative real-time PCR (qPCR) analysis

qPCR was performed to illustrate the expression of *LPL2* using primer LPL2-RT-F/R. SYBR Premix ExTaq (Takara Bio, Inc., Shiga, Japan) and the MX 3050 qPCR System (Stratagene, La Jolla, CA) were used according to the manufacturers’ instructions. The thermal cycling conditions were: 95 °C for 30s, 40 cycles of 95 °C for 15s, and 60 °C for 30s, in a total volume of 20 µl. We used the transcription factor eEF (*LOC_Os03g08020*) as an endogenous control. The specific primers for different genes are listed in Supplementary Table S1. All experiments were repeated at least three times.

### Observation of actin cytoskeleton

The detailed procedure of actin staining was performed as described previously (Frank and Smith, 2002; Panteris *et al.*, 2007). Juvenile leaves of ~2*–* 4cm were cut carefully into 1 cm-long strips, and then prefixed with 200 µM m-maleimidobenzoyl-N-hydroxyl-succinimide ester (MBS) in PEM buffer (50mM PIPES, 5mM EGTA, 2mM MgSO4, pH 6.8), plus 2% (v/v) DMSO and 0.05% (v/v) Triton X-100, for 30min in the dark. Subsequently, MBS solution was removed and replaced by actin ﬁxation solution of 4% (w/v) paraformaldehyde (PFA) in the PEM buffer with 0.1% Triton X-100 and 2% glycerol for 1h. Then the epidermal sheets were rinsed with PEM buffer and extracted in 1% Triton X-100 and 5% DMSO in PEM for 5min, with three repeats. Finally, the 1 cm-long thin strips of leaf tissues were placed into 10% Alexa-Fluor 488 phalloidin dilution (Life Technologies) and stained for 2*–*4h at 37 °C in the dark. All images were analyzed using a confocal microscope (Olympus FluoView FV1000 MPE).

### Yeast two-hybrid assay (Y2H)

The full-length cDNA of LPL2 was ampliﬁed with primers LPL2-Y2H, and then was inserted into GAL4 DNA binding-domain vector pGBKT7 (BD) and trans-activation domain vector pGADT7 (AD), respectively. The full-length cDNA of LPL3 was also cloned into the AD and BD vector with primer LPL3-Y2H. Then, these two groups of plasmids were transformed into yeast strain Y2HGold to culture and observe according to the manufacturer’s manual (Clontech). These experiments were repeated three times. All primers used are listed in Supplementary Table S1.

## Results

### Identiﬁcation and phenotypic analysis of lpl2-1 mutant

**Fig. 1.**
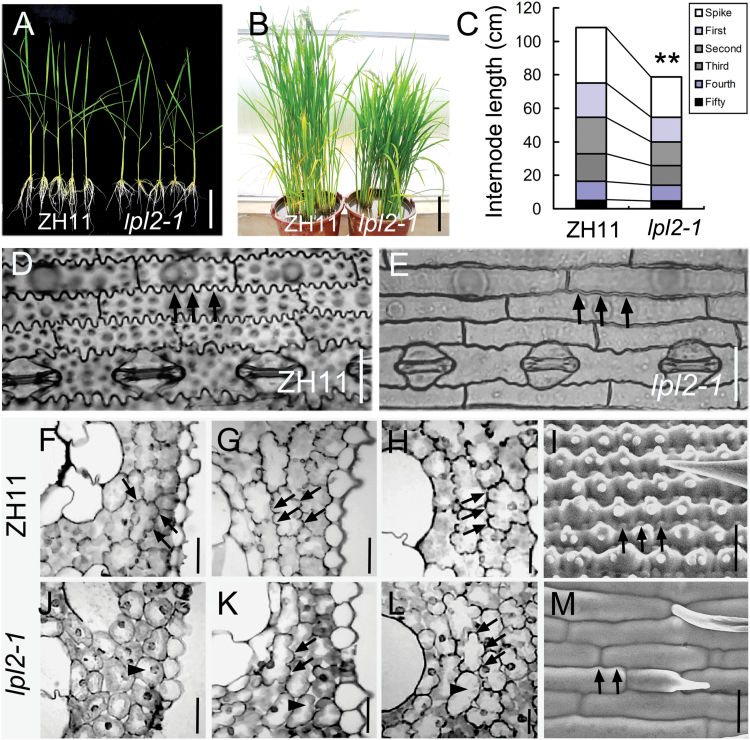
Phenotypes of ZH11 and *lpl2-1*. (A) 28-day-old seedings of ZH11 and *lpl2-1*. Bar, 2cm. (B) Plants of ZH11 and *lpl2-1* at the heading stage. Bar, 15cm. (C) Average internode length. The data presented the mean values (*n*=30); **, *P*<0.01 (Student’s *t*-test). (D, E) Epidermis of ZH11 and *lpl2-1* on abaxial leaves. Epidermal pavement cells of ZH11 with interlocking marginal lobes (arrows); *lpl2-1* epidermal PC lobes were less pronounced lobes (arrows). Bars, 20 μm. (F*–*I), ZH11. (J*–*M), *lpl2-1*. Mesophyll cell profiles of rice leaves on transverse sections of third (F, J), fifth (G, K) and flag leaf (H, L). (I) Scanning electron microscopy (SEM) image of stem epidermal cells with interlocking lateral lobes in ZH11. (M) SEM image of stem epidermal cells with less bulged lobes in *lpl2-1*. Arrows indicate lobes; arrowheads show oval-shaped mesophyll cells. Bars, 20μm. (This figure is available in colour at *JXB* online.)

**Fig. 2.**
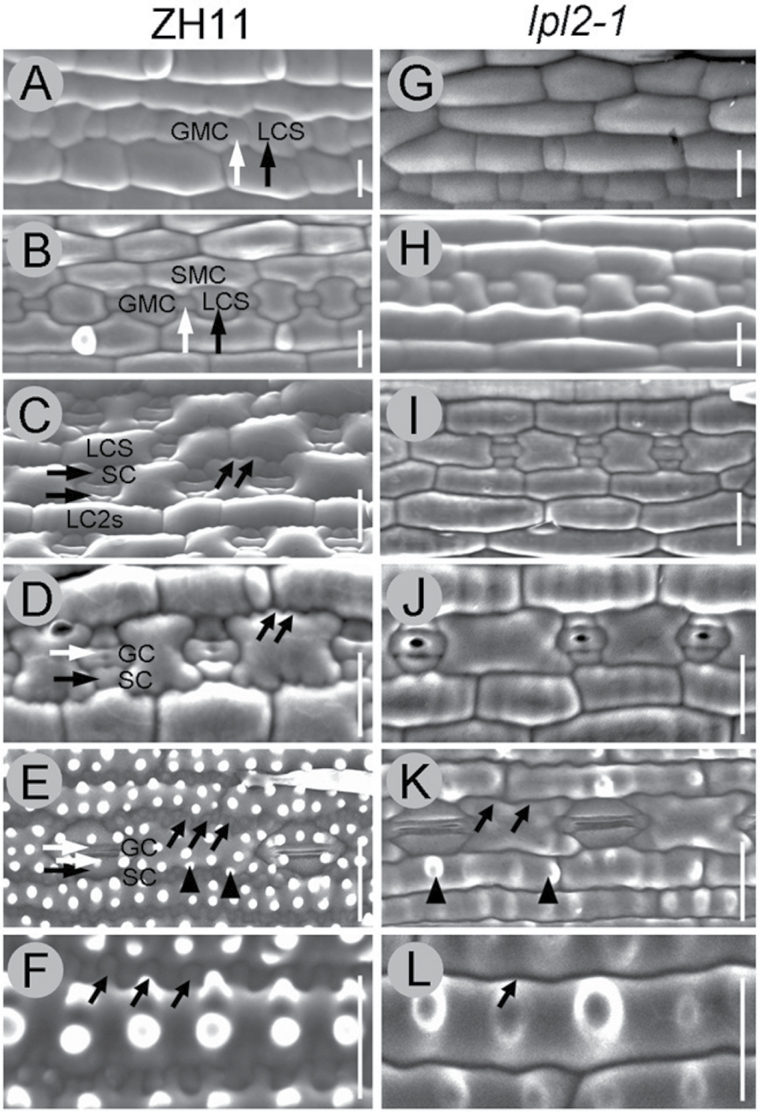
Morphogenic development of stomata and lobes in the abaxial leaf blade of rice. All images were continuously observed by SEM. (A*–*F) ZH11. (G*–*L) *lpl2-1*. (A, G) GMCs (vertical white arrow) and LCSs (vertical black arrow) were produced by the first asymmetric division. (B, H) SMCs initiated the second asymmetric division. (C, I) SCs (parallel black arrow) and LC2s or LCSs were generated by asymmetric divisions. One GMC and surrounding two SCs formed ‘three-cell complexes’, and lobes appeared (oblique black arrows) in ZH11 instead of *lpl2-1* during this stage. (D, J) GCs (parallel white arrow) were produced by symmetrical division and PCs were further extruded. (E) Mature stomata and PCs with waved lobes were obviously observed and abundant papillae appeared in ZH11 (black arrowheads). (F) Amplified mature PCs with elongated and serrated lobes (oblique black arrows). (I*–*L) *lpl2-1* showed epidermal PCs with gentler lobes (oblique black arrow) and decreased papilla (black arrowheads). Bars, 20 μm. LCSs, large long cells in stomata file; SMCs, subsidiary mother cells; LC2s, flanking stomata row long cells.

To explore the genetic and molecular mechanisms that control epidermal cell morphogenesis in rice leaves, a genetic screen for mutants was conducted with altered leaf epidermis morphology. A recessive mutant, *less pronounced lobe epidermal cell2-1* (*lpl2-1*), which exhibits less pronounced and smooth epidermal PCs, was screened from a rice T-DNA insertion mutant library in T1 progeny. This phenotype was stable, but it did not link with the T-DNA insertion. To analyze the function of the *LPL2* gene, the mutant lines without T-DNA were isolated from the T2 progeny, and were backcrossed with wild-type (WT) ‘zhonghua11’ (ZH11, *Oryza sativa*) for three generations in succession. After purification, the height of the aerial part and the root length in *lpl2-1* were shorter than that of ZH11 during early seedling growth (Fig. 1A; Supplementary Fig. S1). During the heading period, *lpl2-1* was also decreased in height because all the internodes of the mutant were shorter than those of ZH11 (Fig. 1B, C). At the mature stage, *lpl2-1* had shorter panicle than ZH11 (Supplementary Fig. S2). Notably, clearer and smoother epidermal PCs were found in *lpl2-1* (Figs 1D, E, 2E, K). In the ZH11 leaf blade epidermis, PCs have lobes along their lateral margins, which interlock with adjacent PCs (Fig. 1D, arrows). Nevertheless, *lpl2-1* had epidermal PCs with smooth marginal lobes or even reduced lobes (Fig. 1E, arrows). Additionally, the phenotype of the less pronounced lobes was also observed in mesophyll cells, as well as stem cells, in the mutant (Fig. 1F–M). The transverse sections of mesophyll cells of ZH11 showed a wreath shape, whereas *lpl2-1* displayed an oval shape in the third leaf, fifth leaf, and flag leaf (Fig. 1F–H, J–L). These phenotypes suggest that the lobe-forming cell types substantially differ between *lpl2-1* and ZH11 during the same development period.

### lpl2-1 exhibits severe defects in epidermal PC lobe development

To investigate the remarkable difference in the epidermal PC lobes between *lpl2-1* and WT in more detail, the development process of leaf epidermal PCs was compared following the period of stomatal development. In general, rice stomatal development requires two asymmetric, and one symmetric, cell divisions in rice leaves (Fig. 2A–E) (Kamiya *et al.*, 2003; Luo *et al.*, 2012). During the two asymmetric divisions of stomata, epidermal PC morphogenesis of *lpl2-1* was consistent with that of ZH11 (Fig. 2A, B, G, H). After completing the two asymmetric divisions, stomata are formed by ‘three-cell complexes’ (Fig. 2C, I). At this phase, following the elongation and broadening of PCs, many lobes are initiated from the epidermal PC margins in ZH11 (Fig. 2C). Subsequently, guard mother cells (GMCs) divide symmetrically to produce two guard cells (GCs), and the marginal lobes of PCs become more extended (Fig. 2D). However, during this period, no obvious lobes emerged at the edge of epidermal PCs in *lpl2-1* (Fig. 2I, J). At maturity, the stomatal complex is composed of two dumbbell-shaped GCs that are flanked by two triangular-shaped subsidiary cells (SCs), and epidermal PCs with serrated marginal lobes are covered with numbers of papillae in ZH11 leaves (Fig. 2E, F). Nevertheless, *lpl2-1* epidermal PCs revealed a smooth epidermis, which showed less pronounced lobes and little uniform distribution of papillae (Fig. 2K, L). Therefore, *LPL2* was involved in the lobe formation during the leaf epidermis morphogenesis of rice.

### lpl2-1 also showed abnormal stomatal pattern

**Fig. 3.**
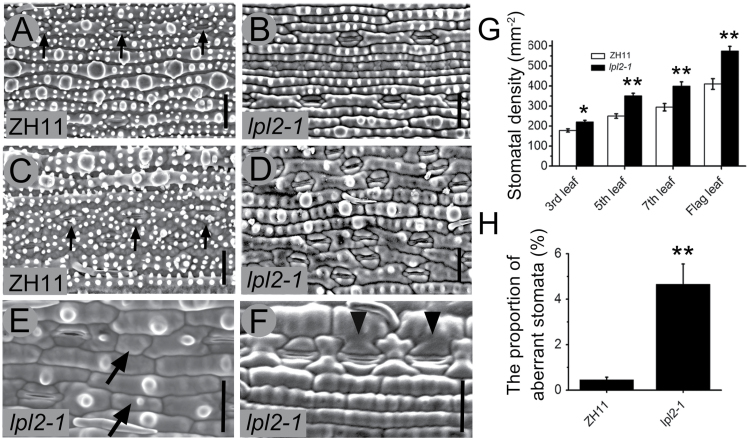
*lpl2-1* showed increased stomatal density and aberrant stomata. SEM images of the third abaxial leaf in ZH11 (A), and *lpl2-1* (B). SEM images of the abaxial side of flag leaf in ZH11 (C) and *lpl2-1* (D). Vertical arrows present stomata. (E, F) SEM images of aberrant stomata in *lpl2-1*. Diagonal arrows indicate GMC-like cells. Arrowheads indicate aberrant SCs. Bars, 20 μm. (G) Stomatal density of ZH11 and *lpl2-1*, *n*>100. (H) The proportion of aberrant stomata in ZH11 and *lpl2-1*, n>6000. Error bars represent SD; *, *P*<0.05; **, *P*<0.01 (Student’s *t*-test).

With increased phyllotaxis, the stomatal density was gradually enhanced in both ZH11 and *lpl2-1*. In contrast, statistical analysis revealed that the stomatal density of *lpl2-1* was considerably higher than that of ZH11 in the same phyllotaxis (Fig. 3A–D, G). Some abnormal stomata were also observed in *lpl2-1*, and the proportion of aberrant stomata in *lpl2-1* was significantly higher than that of ZH11 (Fig. 3E, F, H). GMC-like cells that lose the ability to continuously divide were occasionally observed in fully developed juvenile and adult leaves of *lpl2-1* (Fig. 3E, diagonal arrows). Some aberrant SCs were produced due to the abnormal cell division in subsidiary mother cells (SMCs) (Fig. 3F, arrowheads). These results indicated that *LPL2* affects morphological traits associated with leaf stomatal density and distribution pattern.

### Map-based cloning of LPL2 gene

**Fig. 4.**
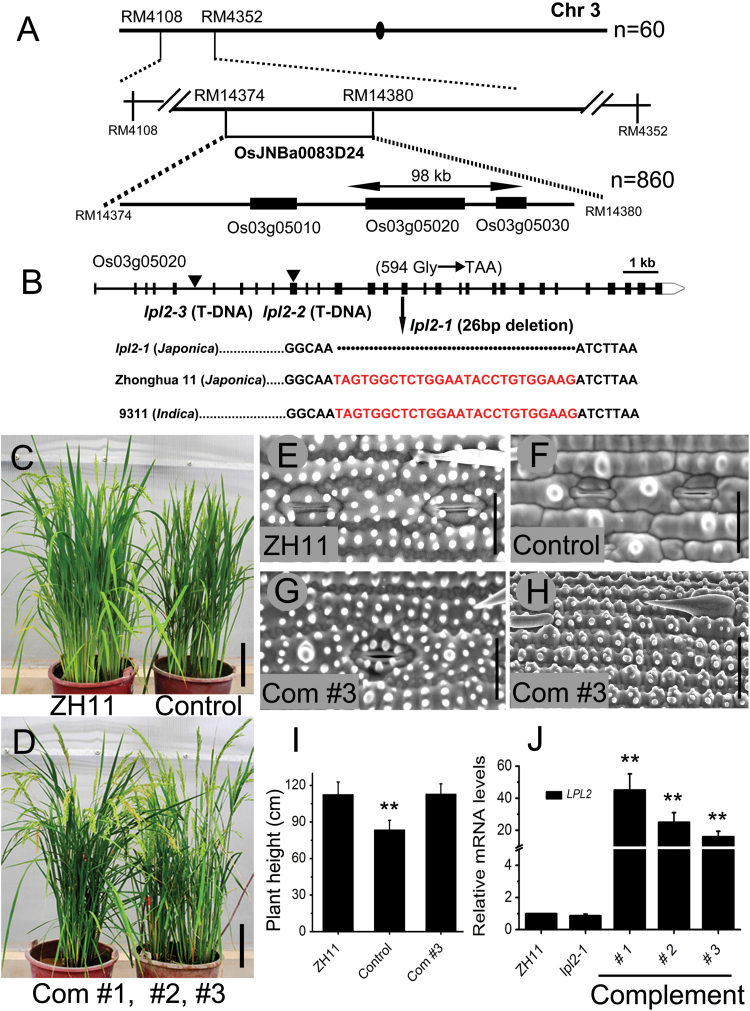
Map-based cloning of *LPL2* and complementation test. (A) Fine mapping of the *LPL2*. *LPL2* was localized within BAC clone OsJNBa0083D24 on chromosome 3. N represents sample size. (B) Genomic structure of *LPL2*. Arrowheads represent T-DNA insertion localization of *lpl2-2* and *lpl2-3*. Vertical arrow shows 26bp deletion of *lpl2-1* at the 16th exon. Boxes indicate exons and lines between the boxes indicate introns. (C) ZH11 and control transgenic plants. (D) Plants of three complemented lines (com #1, #2 and #3). Bar, 15cm. SEM images of epidermis on the abaxial leaves of ZH11 (E), control (F), and the com #3 (G). (H) SEM image of stem epidermis in the com #3. Bars, 20 μm. (I) Plant height of ZH11, control and com #3, *n*=15. (J) Relative mRNA levels of *LPL2*. The experiment was repeated at least three times. Error bars represent SD; **, *P*<0.01 (Student’s *t*-test). (This figure is available in colour at *JXB* online.)

All F1 plants of reciprocal crosses between *lpl2-1* and ZH11 were identical to WT, whereas in the self-pollinated F2 population, ZH11 and aberrant epidermal PC phenotypic plants showed a ratio of ~3:1 (ZH11:*lpl2-1*=260:75), suggesting that *lpl2-1* was most likely caused by a single recessive mutation. To clone the causal gene for *LPL2*, *lpl2-1* was cross fertilized with ‘9311’, a WT polymorphic *indica* rice, to construct a small scale mapping population of backcross 9311 F1 (BC3F1). After self-pollination, BC3F2 mapping population was constructed successfully. The candidate gene locus was fine mapped in a ~98-kb region between two known molecular markers (RM14374 and RM14380) in chromosome 3 by map-based cloning (Fig. 4A). Subsequently, 14 genes within this range were sequenced, and a deletion of 26 base pairs compared with ZH11 and 9311 was detected and ascertained in *LOC_Os03g05020* (full-length gene nearly 16kb). However, when cloning and sequencing the *LOC_Os03g05020* cDNA based on three independent PCR products, the full-length cDNA was composed of 30 exons, and a 3862bp full-length coding region, instead of the predicted mRNA, which comprised 27 exons and 3555bp full-length cDNA from Rice GE (http://signal.salk.edu/cgi-bin/RiceGE/) (Fig. 4B). Therefore, the new cDNA sequences, including the 9th, 11th, and 12th exons, were supplemented and completed in *LOC_Os03g05020* cDNA (Supplementary Fig. S3). Based on the new cDNA sequence, we found that the deletion mutation in *lpl2-1* led to a frameshift mutation in the 16th exon, and the re-translated 594th glycine was turned into a stop codon (Fig. 4B). *LOC_Os03g05020* encodes a putative PIR protein, which is a subunit of the SCAR/WAVE complex, and is homologous to the Arabidopsis PIR/SRA1 and human PIR121/SRA1 protein (Kobayashi *et al.*, 1998; Basu *et al.*, 2004). It was named *LOC_Os03g05020* coinciding with *LPL2* in rice. LPL2 shared the highest amino acid similarity with BRK2 in maize (93% identity), displayed 74% sequence similarity to PIR in Arabidopsis, and identified 27% with human PIR121 (Supplementary Fig. S4). The sequence similarity alignment assay showed that *LPL2* is somewhat highly conserved throughout the plant kingdom.

### LPL2 is responsible for lobe deficiency in leaf and stem PCs as shown by genetic and transgenic evidence

To further confirm the candidate gene, a complementation assay was performed by overexpressing *LPL2* (*OE-LPL2*) with the maize ubiquitin promoter in *lpl2-1*. Twenty individual lines from complemented transgenic line #1 (com #1) to com #20 of *OE-LPL2/lpl2-1* were completely rescued, including the reduced plant height and aberrant epidermal cell phenotypes, whereas the control lines with the empty plasmid failed to restore normal phenotypes (Fig. 4C–I). Additionally, the expression level of *LPL2* was examined in independent transgenic lines of *OE-LPL2/lpl2-1* qPCR, and found the relative mRNA level to be 12*–*45 times higher than that of ZH11 (Fig. 4J). Concurrently, ten individual transgenic lines of *OE-LPL2/*ZH11 were not substantially different from ZH11 in their phenotype. These results confirmed that the locus *LOC_Os03g05020* represents the *LPL2* gene.

**Fig. 5.**
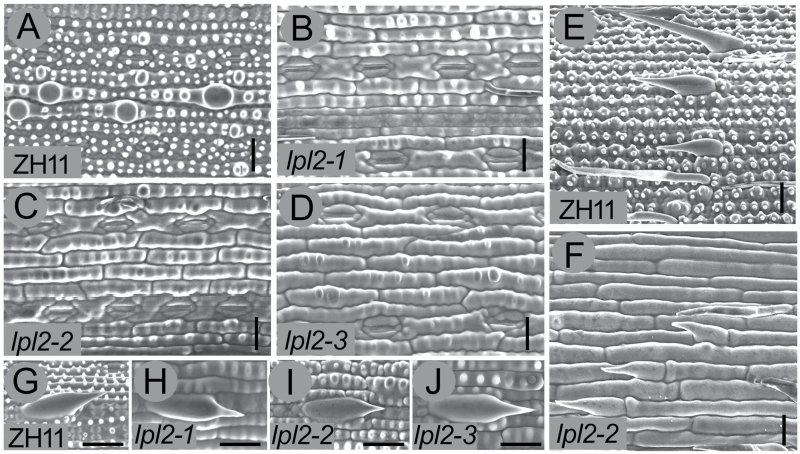
Epidermis of ZH11, *lpl2-1*, *lpl2-2*, and *lpl2-3*. SEM images of the abaxial leaves of ZH11 (A), *lpl2-1* (B), *lpl2-2* (C) and *lpl2-3* (D). SEM images of the stem epidermis in ZH11 (E) and *lpl2-2* (F). Bars, 20 μm. SEM images of the leaf prickle hair in adaxial leaf of ZH11 (G), *lpl2-1* (H), *lpl2-2* (I) and *lpl2-3* (J). Bars, 20 μm.

Furthermore, to verify the function of *LPL2* in epidermal cell morphogenesis, two *LPL2* allelic mutants, designated *lpl2-2* and *lpl2-3* (T-DNA lines from Rice Mutant Database, China), were isolated (Fig. 4B). Interestingly, the abnormal leaf and stem epidermis, decreased plant height, short root, and short panicle phenotypes in *lpl2-2* and *lpl2-3* were indistinguishable from those of the *lpl2-1* mutant (Fig. 5A–F). Consistently, no obvious phenotypic difference in leaf prickle hairs was found between *lpl2* mutants and ZH11 (Fig. 5G–J). More notably, the seedlings and epidermis of *lpl2-1/-2* and *lpl2-1/-3*, which were F1 hybrids between *lpl2-1* and *lpl2-2*, *lpl2-1* and *lpl2-3*, respectively, were identical to that of *lpl2-1* (Supplementary Fig. S5). Therefore, the genetic analysis indicated that these three *lpl2* mutants were controlled by the same gene. Altogether, the genetic and transgenic evidence demonstrated that loss-of-function of *LPL2* results in an abnormal epidermis and other phenotypes.

### Overexpression of LPL2 can partially rescue the distorted trichomes in the Arabidopsis pir mutant

**Fig. 6.**
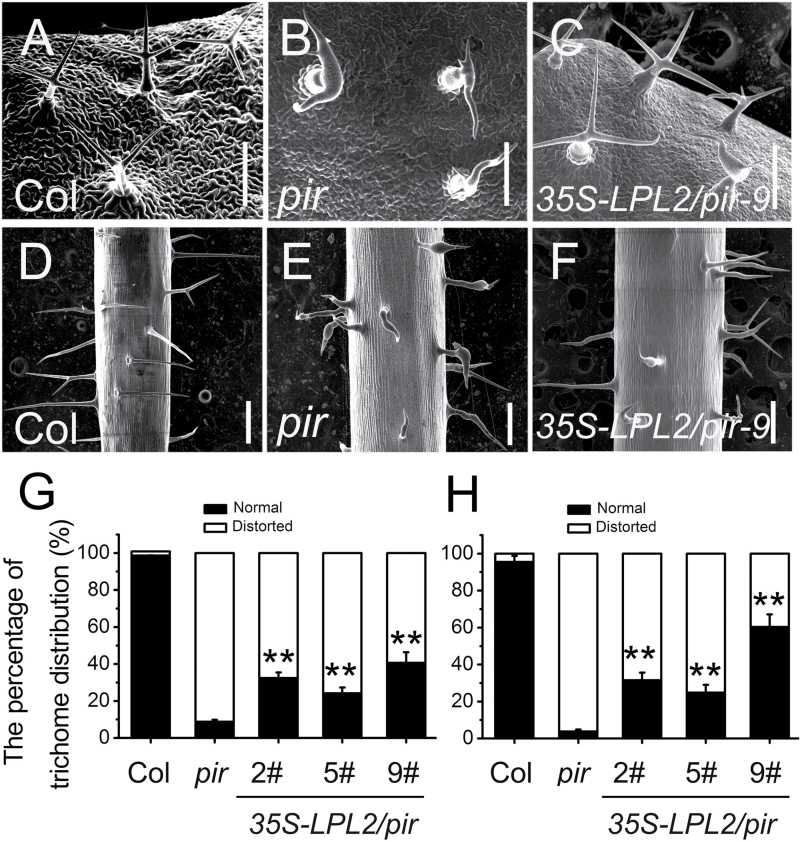
Overexpression of *LPL2* could partially complement phenotypes of distorted trichomes in Arabidopsis *pir*. SEM images of trichomes on the adaxial leaf of Col (A), *pir* (B) and *35S-LPL2/pir-9#* (C). Bars, 400 μm. SEM images of trichomes on the stem of Col (D), *pir* (E) and *35S-LPL2/pir-9#* (F). Bars, 0.5cm. Panel A shows leaf trichomes on 2-week-old Col with three-branch shape; panel B shows *pir* exhibited defective trichomes; panels D, E show stem trichomes are straight in Col, but distorted in *pir*, respectively. The percentage of trichomes with normal or abnormal morphology on the leaf epidermis (G) and stem epidermis (H). *n*>100. Error bars represent SD. **, *P*<0.01 (Student’s *t*-test).

Basu *et al.* (2004) revealed that Arabidopsis PIROGI encodes a homolog human protein PIR121/SRA1, and their functions could interchange during leaf epidermal cell development. It is reasonable to presume that functions of PIR and its homolog protein LPL2 are functionally interchangeable because of their conserved amino acid sequence (Supplementary Fig. S4). To verify this speculation, a *pir* mutant induced by a T-DNA insertion in Arabidopsis (SALK_106757), which shows various defective trichomes on leaves and stems compared to WT, was ordered (Fig. 6A, B, D, E; Supplementary Fig. S6). Whereas, when the full-length cDNA of *LPL2* was overexpressed into *pir* plants, driven by a cauliflower mosaic virus 35S promoter (*35S-LPL2*), most of the T1 generation plants could be partially rescued in terms of the distorted leaf and stem trichomes (Fig. 6G, H). Moreover, the more strongly rescued phenotype was found to be *35S-LPL2/pir*-9#. Statistical analysis showed that the percentage of normal trichomes was ~40% and 60% on the leaf and stem epidermis of *35S-LPL2/pir*-9#, respectively (Fig. 6C, F–H). Partial complementation of mutant phenotypes in these transgenic lines could be inherited after self-fertilization. These results indicated that the functions of LPL2 and PIR are conserved, but could not be completely interchanged.

### The aberrant distribution of local actin filaments leads to less pronounced lobes of epidermal PCs in lpl2-1

**Fig. 7.**
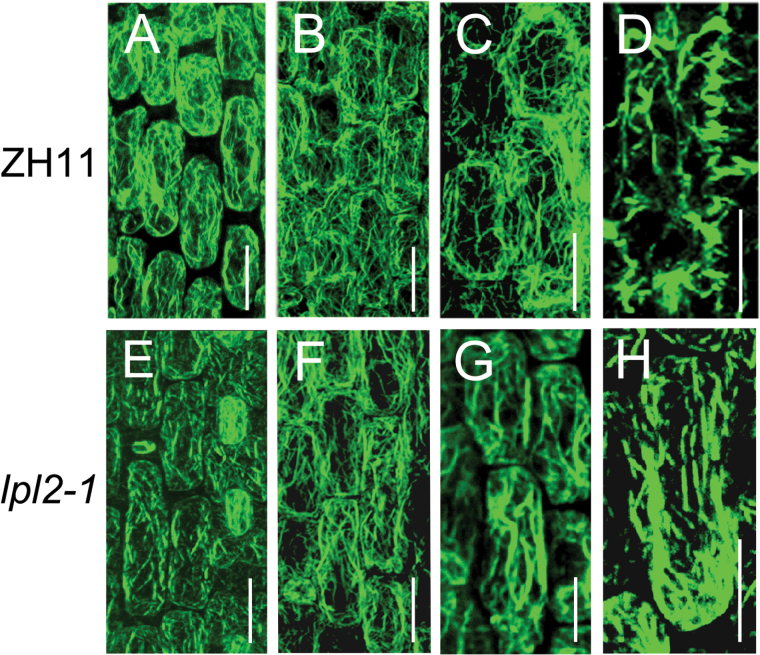
The aberrant distribution of localized actin filaments contributed to smooth lobes in *lpl2-1* epidermal PCs. (A*–*H) Images of confocal-laser-scanning microscope showed epidermal PCs in young leaves by Alexa Fluor 488-phalloidin staining. (A*–*D) Epidermal PC lobe development and distribution of ZH11. (E*–*H) Epidermal PC lobe development and distribution of *lpl2-1*. Plants, with heights of 2*–*3cm (A, B, E, F), 5–6cm (C, G) and ~8cm (D, H) were selected for staining, respectively. Three independent plants were analyzed. Bars, 20 μm.

Combined with LPL2 function in encoding a subunit of the WAVE complex, and previous studies on maize and Arabidopsis epidermis, *LPL2* associates with actin cytoskeleton morphogenesis in rice (Frank and Smith, 2002; Frank *et al.*, 2003; Basu *et al.*, 2004; Brembu *et al.*, 2004). To assess the epidermal PC morphologic abnormality underlying F-actin rearrangement in rice leaves, the ﬁxed cells of ZH11 and *lpl2-1* were observed by staining with Alexa Fluor 488-phalloidin. Prior to the initiation of PC lobes, there were no significant differences between ZH11 and *lpl2* mutants (Fig. 7A, B, E, F). Following the expansion of epidermal PCs, pronounced cortical F-actin aggregations/patches enriched at the cell margin sites of lobe emergence and extension, and eventually formed serrated lateral lobes in ZH11 epidermal PCs (Fig. 7C, D; Supplementary Fig. S7). In the *lpl2-1* epidermal PC margin, however, lesser abundant and prominent cortical F-actin patches were observed (Fig. 7G, H; Supplementary Fig. S7). As expected, these results highlighted that the epidermal PC lobe deficiency in *lpl2-1* is a result of disorganization of the actin cytoskeleton. These results indicated that *LPL2* is a functional SCAR/WAVE protein, which plays an evolutionarily conserved role in controlling epidermal cell morphogenesis in rice, as well as in maize and Arabidopsis.

### lpl3-1 exhibits deficient phenotypes similar to lpl2-1 in leaf and stem epidermis arrangement

**Fig. 8.**
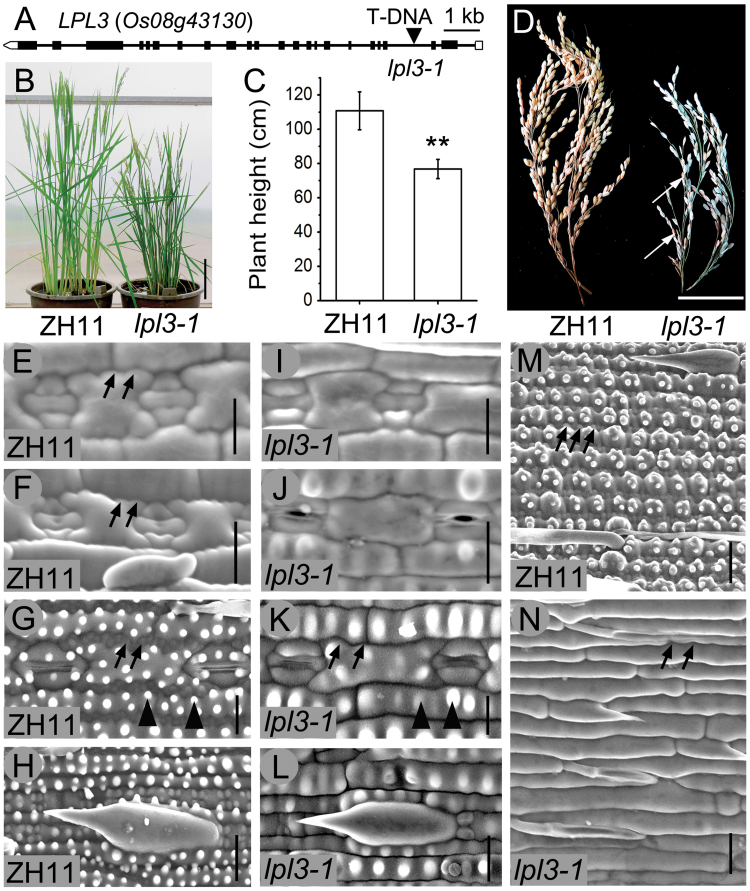
Phenotypic analysis of *lpl3-1*. (A) The structure diagram of *LPL3* gene; boxes indicate exons and lines between the boxes indicate introns. Arrowhead represents T-DNA insertion location of *lpl3-1*. (B) Plants of ZH11 and *lpl3-1* at the heading stage. Bar, 15cm. (C) Plant height of ZH11 and *lpl3-1*, *n*=30. Error bars represent SD, **, *P*<0.01 (Student’s *t*-test). (D) Spikes of ZH11 and *lpl3-1*. Poor fertility in *lpl3-1*. Oblique white arrows indicate plump seeds. Bar, 5cm. SEM images of lobe appearance and development in leaf abaxial epidermis of ZH11 (E*–*G) and *lpl3-1* (I*–*K). (G, K) Mature PCs with serrated lobes and numerous papillae (black arrowheads) in ZH11 and gentler lobes and reduced papilla (black arrowheads) in *lpl3-1*. SEM images of the leaf prickle hair in ZH11 (H) and *lpl3-1* (L). SEM images of the stem epidermis in ZH11 (M) and *lpl3-1* (N). Oblique black arrows indicate lobes. Bars, 20 μm. (This figure is available in colour at *JXB* online.)

Bioinformatics analysis revealed another subunit of the WAVE complex, the NAP1-like protein (LOC_Os08g43130) in rice (http://www.ebi.ac.uk/Tools/sss/ncbiblast/), which is a homolog of BRK3 in maize and NAP1 in Arabidopsis. We identified its T-DNA insertion mutants (ACNB06, France), which exhibited deficient phenotypes similar to those of *lpl2-1*, including less pronounced and smooth epidermal PCs, moderate dwarfism, and poor fertility (Fig. 8A–N), and named *lpl3-1*. The development process of leaf PC lobes in *lpl3-1* was consistent with that of *lpl2-1*. During the ‘three-cell complexes’ stage and guard mother cell symmetrical division phase, many lobes appeared and gradually expanded from the epidermal PC margins in ZH11, but not in *lpl3-1* (Fig. 8E, F, I, J). During the period of stomata and PC maturation, *lpl3-1* leaf epidermal PCs exhibited less pronounced lobes and strong reduction in papillae formation compared to those in ZH11 (Fig. 8G, K). In addition, stem PC lobes in *lpl3-1* were considerably smoother than those in ZH11 (Fig. 8M, N). The morphology of the leaf prickle hairs was not substantially different between *lpl3-1* and ZH11 (Fig. 8H, L). F-actin disorganization of the leaf epidermal PC lobes in *lpl3-1* was scarcely different from that of *lpl2-1* using Alexa Fluor 488-phalloidin staining (Supplementary Fig. S8). Therefore, the defect of epidermal PC lobes in *lpl3-1* was considered to be caused by the abnormal rearrangement of local actin filaments. The full-length *LPL3* cDNA was cloned and sequenced; it consisted of 22 exons and a 4080-bp coding region (Fig. 8A). LPL3 protein sequences were conserved both in plants and animals. It shared 88.3%, 66.2%, and 17.3% amino acid similarity to homologous proteins ZmBRK3, AtNAP1, and HsNAP1, respectively (Supplementary Fig. S9). These results suggested that *LPL3* also plays an important role in regulating epidermal cell morphogenesis in rice, and it is another functional SCAR/WAVE protein.

### LPL2 directly interacts with LPL3 in vitro

**Fig. 9.**
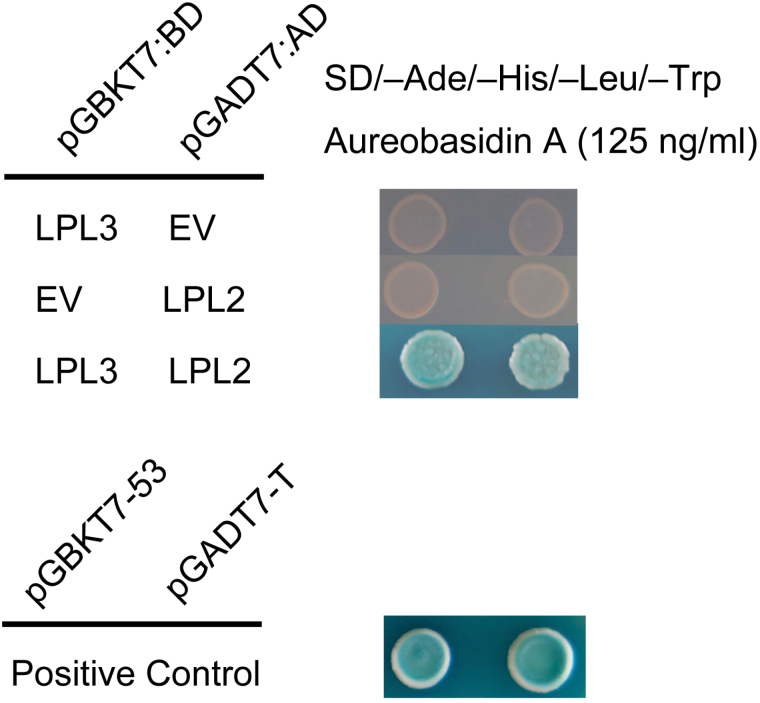
LPL2 interacts with LPL3 in the Y2H system. EV, empty vector. pGBKT7-53/pGADT7-T was used as a positive control.

Based on the interaction of PIR and NAP1 proteins in previous investigations, it was speculated that LPL2 and LPL3 proteins could directly interact (Basu *et al.*, 2004; Chen *et al.*, 2010). Therefore, a yeast two-hybrid (Y2H) analysis in yeast strain Y2HGold was conducted, with LPL3 expressed as a DNA binding domain (BD) fusion protein, and LPL2 as a transactivation domain (AD) fusion protein. The interaction of LPL2-AD and LPL3-BD was observed and confirmed (Fig. 9). These data indicated that LPL2 directly interacts with LPL3 *in vitro*, and implied that both of them belong to the SCAR/WAVE complex in rice.

## Discussion

In this work, a mutant with smooth epidermal PC lobes was screened using forward genetics. Molecular genetic studies indicated that the gene *LPL2* in rice is homologous to *BRK2* in maize (Poaceae family). This work demonstrated that *LPL2* encodes a PIR-like protein, a subunit of the functional SCAR/WAVE complex involving in F-actin nucleation in Arabidopsis, rice, maize, and animals (Frank *et al.*, 2003; Basu *et al.*, 2004; Patel *et al.*, 2008; Chen *et al.*, 2010; Bai *et al.*, 2015; Facette *et al.*, 2015).

In different species, a deﬁciency of the SCAR/WAVE regulatory complex always results in epidermal morphological changes. In Arabidopsis, mutations in all elements of well-known *SCAR/WAVE* complex display a similar phenotype, which shows distorted trichome morphology due to disorganized distribution of F-actin patches (Basu *et al.*, 2004; Brembu *et al.*, 2004; Li *et al.*, 2004; Djakovic *et al.*, 2006; Le *et al.*, 2006). In rice, this study found that *lpl2-1* showed evident epidermal PC lobe defects in leaves due to impaired actin filament organization (Figs 1D, E, 7D, H). Recent studies have consistently revealed that TUT1/OsSCAR1/ES1, another subunit of the SCAR/WAVE complex, controls morphological changes in rice leaf trichomes and root epidermal cells by modulating actin polymerization (Bai *et al.*, 2015; Rao *et al.*, 2015). Furthermore, similar to the rice *es-1* mutant, *lpl2-1* showed smoother epidermis and a higher stomatal density than that of WT, suggesting that *LPL2* is closely associated with drought stress tolerance in rice (Rao *et al.*, 2015). The increased stomatal density and smooth epidermis would increase water loss in *lpl2-1* plants. In addition, the less pronounced lobes of PCs in *lpl2* was consistent with leaf epidermal PCs in maize mutant *brk1*, *brk2*, and *brk3*, which lack interlocked lobes in the PC margin because of an altered F-actin arrangement (Frank and Smith, 2002; Frank *et al.*, 2003). Three *lpl2* mutants, in mesophyll cell lobe morphology and leaf hairs, were considerably different from maize *brk1* mutant with normal mesophyll cell lobes, and blunter, shorter hairs (Frank and Smith, 2002; Frank *et al.*, 2003). Altogether, mutants of *lpl2*, and its homologous genes, developed similar phenotypes, suggesting that LPL2 and its homologs have a common mechanism in controlling F-actin nucleation.

Meanwhile, in *lpl2* mutants, higher stomatal density and deformed stomata cells were also observed, implying that *LPL2* might be involved in stomatal development and polarized cell division in the rice leaf epidermis (Fig. 3). While the current understanding of rice gene function in controlling epidermal cell morphogenesis remains limited, studies similar to this with *LPL2*, affecting leaf epidermal morphogenetic patterns and polarized cell division, have been widely explored in maize. *BRK1*, *BRK2*, *BRK3*, *PANGLOSS1* (*PAN1*), *PAN2*, *DISCORDIA1*, *ALTERNATIVE DISCORDIA1*, and *ZmROP2/ZmROP9* play an extremely important role in regulating the SMC asymmetric division and epidermal PC development. Mutations in these genes will give rise to abnormal SCs and PCs (Gallagher and Smith, 1999; Frank and Smith, 2002; Frank *et al.*, 2003; Cartwright *et al.*, 2009; Wright *et al.*, 2009; Humphries *et al.*, 2011; Sutimantanapi *et al.*, 2014). Moreover, Facette *et al.* (2015) recently reported that BRK1 and BRK3 function with PAN1 and PAN2 to promote SMC asymmetric division in maize leaves. During rice plant growth and development, epidermal patterning formed a regular distribution, which is especially similar to maize plants (Liu *et al.*, 2009). Therefore, it was speculated that the analogous genes would exist in rice because of their conserved functions in the regulation of epidermal cell morphogenesis.

Similarly, *LPL3* is a homolog of *BRK3* in maize, and it encodes an NAP1-like protein, a subunit of the SCAR/WAVE complex in Arabidopsis (Frank *et al.*, 2003; Brembu *et al.*, 2004; Deeks *et al.*, 2004; El-Assal *et al.*, 2004; Li *et al.*, 2004). *lpl3-1* was isolated using a reverse genetic approach; it also showed almost identical epidermal PC features among rice *lpl2* and three maize *brk* mutants (Frank and Smith, 2002; Frank *et al.*, 2003). The similar phenotypes of *lpl2* and *lpl3* indicate that the SCAR/WAVE complex might function via the interaction between LPL2 and LPL3. In Arabidopsis, aberrant trichomes were observed in the *nap1* mutant, similar to the phenotypes of *pir*, because the F-actin cytoskeleton showed a disturbed spatial orientation (Brembu *et al.*, 2004; Deeks *et al.*, 2004; Li *et al.*, 2004). Similarly, blunted tips of the trichomes were observed in rice *tut1/scar1* mutant, although the shape of leaf prickle hairs had no distinction between *lpl2*, *lpl3*, and WT (Bai *et al.*, 2015; Rao *et al.*, 2015). These results indicated that deficiency of the NAP1-like protein in different species principally leads to an abnormal morphology in the leaf epidermal PCs and trichomes, suggesting that the functions of LPL3 are conserved with those of BRK3 and NAP1 in plants. However, the reason for trichome trait variation across different SCAR/WAVE complex subunit mutants in rice is not known. A plausible explanation is that the mechanism controlling the morphology and development of PCs and trichomes may be not exactly identical in these different subunits. For instance, blunted trichomes were found in rice mutant *scar1*, but the abnormal phenotype of PC lobes was not significant (Bai *et al.*, 2015; Rao *et al.*, 2015). In this study, smooth PC lobes rather than blunted trichomes were observed in *lpl2* and *lpl3*. Therefore, the diverse functions of different SCAR/WAVE elements in rice involved in epidermal cell morphogenesis need to be further elucidated.

In most dicot leaf epidermises, the formation of lobes and interdigitation is a significant characteristic (Szymanski, 2014). Compared to unlobed cells, one of the important functions of interlocked cells is likely to be increased mechanical strength (Szymanski, 2014). Having similar role(s) in dicots, and in the leaf epidermis of monocots, the waviness of epidermal PCs might also increase its toughness and water tightness, which can enhance crop resistance and improve yield. Thereby, the gentler leaf epidermal PC lobes in *lpl2* and *lpl3* might lead to reduced adhesive properties of PC walls, and decreased stress tolerance (Panteris and Galatis, 2005; Szymanski, 2005). Furthermore, the defects of these F-actin aggregations/patches could be primarily responsible for PC lobe formation and development failure in *lpl2* and *lpl3*. Apart from the observations made with *lpl2* and *lpl3*, maize *brks*, Arabidopsis *pir*, *nap1*, and *brk1*, also showed abnormalities in F-actin organization (Frank and Smith, 2002; Brembu *et al.*, 2004; Le *et al.*, 2006). Therefore, the actin patches seem to participate in further PC lobe growth and extension. These results support the idea that PCs develop a less wavy pattern in the above mutants mainly due to the absence of F-actin bundles at the tips of the initiated lobes (Frank *et al.*, 2003; Panteris and Galatis, 2005).

The results of this study revealed that LPL2 and LPL3 are important components for assembling the SCAR/WAVE complex in rice. A yeast two-hybrid assay proved that LPL2 directly interacts with LPL3, consistent with previous findings that NAP1/GNARLED and PIR/KLK/SRA1 can directly interact in plants and animals (Basu *et al.*, 2004; El-Assal *et al.*, 2004; Uhrig *et al.*, 2007; Chen *et al.*, 2010). Furthermore, crystal structural analysis of the SCAR/WAVE complex reveals a compositional process in which NAP1 and PIR/SRA1 extensively interact to create a dimer. This dimer combines with the other tripolymer, which is composed of ABI, SCAR, and HSPC300/BRK1, forming a pentameric SCAR/WAVE regulatory complex in animals (Chen *et al.*, 2010). This complex is activated by Racs/Rops, and promotes ARP2/3 complex actin nucleation (Eden *et al.*, 2002; Steffen *et al.*, 2004; Uhrig *et al.*, 2007; Lebensohn and Kirschner, 2009). Moreover, in maize, the physical interaction between ZmROP2 and ZmSRA1/BRK2 demonstrated that ROP regulates SCAR/WAVE function in SMCs, consistent with prior reports that suggest interactions between Rop/Rac and PIR/SRA1 in plants and animals (Facette *et al.*, 2015). Taken together, this work revealed that LPL2 and LPL3 may interact to create a dimer of the SCAR/WAVE complex, which might be activated by one member of the OsRop/Rac family, and play a crucial role in regulating epidermal cell morphogenesis in rice.

It is worth noting that Uhrig *et al.* (2007) found a conserved molecular mechanism showing that actin-nucleation depends on signaling from Rops/Racs-WAVE-ARP2/3 and is crucial for controlling epidermis morphogenesis in both plants and animals (Eden *et al.*, 2002; Bogdan and Klambt, 2003; Kunda *et al.*, 2003; Frank *et al.*, 2004; Basu *et al.*, 2005; Lebensohn and Kirschner, 2009). Bai *et al.* (2015) proved that rice *TUT1/SCAR1* can activate ARP2/3 to promote actin nucleation and polymerization *in vitro*. Hence, *LPL2* and *LPL3* should be involved in the signaling pathway of OsRacs-SCAR/WAVE-OsARP2/3. However, which other subunits are a part of the SCAR/WAVE complex, and how they coordinate to work in rice, are still unknown. Consequently, further investigation might focus on the primary signaling pathway of the cytoskeleton, which plays an essential role in epidermal cell morphogenesis and basic plant cell shape by regulating the polymerization of F-actin in rice.

## Supplementary data

Supplementary data are available at *JXB* online.

Table S1. Primers used in this study.

Figure S1. Plant height and root length of ZH11 and *lpl2-1* at the seedling stage.

Figure S2. Phenotype of spikes in ZH11 and *lpl2-1*.

Figure S3. Supplement of *LPL2* cDNA sequence.

Figure S4. Amino acid alignment of Arabidopsis PIR, rice LPL2, maize BRK2 and human PIR121.

Figure S5. Seedlings and leaf abaxial epidermis of ZH11, lpl2-1, *lpl2-1/-2* and *lpl2-1/-3*.

Figure S6. Plant and trichomes of adaxial leaves in Col and *pir*.

Figure S7. The proportion of cells showing actin patches in ZH11 (Fig. 7C) and *lpl2-1* (Fig. 7G).

Figure S8. Analysis of actin cytoskeletal structures in ZH11 and *lpl3-1* mature leaf epidermal cells.

Figure S9. Amino acid alignment of Arabidopsis NAP1, rice LPL3, maize BRK3 and human NAP1.

Supplementary Data
