# Supplementary material for: Homologs of SCAR/WAVE complex components are required for epidermal cell morphogenesis in rice
Source: J Exp Bot. 2016 Jun 1;67(14):4311–23. doi: 10.1093/jxb/erw214 (PMC5301933; doi:10.1093/jxb/erw214)
Supplement: Supplementary Data [file supp_67_14_4311__index.html]

Homologs of SCAR/WAVE complex components are required for epidermal cell morphogenesis in rice — Homologs of SCAR/WAVE complex components are required for epidermal cell morphogenesis in rice — Supplementary Data 

# Homologs of SCAR/WAVE complex components are required for epidermal cell morphogenesis in rice

## Supplementary Data

Data files

- supplementary\_table\_S1\_figures\_S1\_S9.pdf - Supplementary Data
