# Supplementary material for: Homologs of SCAR/WAVE complex components are required for epidermal cell morphogenesis in rice
Source: J Exp Bot. 2016 Jun 1;67(14):4311–23. doi: 10.1093/jxb/erw214 (PMC5301933; doi:10.1093/jxb/erw214)
Supplement: Supplementary Data [file supp_erw214_supplementary_table_S1_figures_S1_S9.pdf]

Supplemental Data

Supplementary Table S1. Primers used in this study

Primer	Forward primer	Reverse primer	Use
eEF	TTTCACTCTTGGTGTGAAGCAGAT	GACTTCCTTCACGATTTCATCGTAA	qRT-PCR
LPL2-RT	CCTGGAATCATCTCGTGTTATCC	GCTGGGCAGAGTCATTGTAAAG	qRT-PCR
LPL2-2FP	ACTTGATCCTTTGCTGGCAC	TGGGATTCTTGGATAATAGCC	Verify T-DNA
PFRB4-RB		TGCAGGTTCTCTCCAAAT	Verify T-DNA
LPL2-3FP	TCTCCAAGTGATGTGCAAATTC	TGCAGGGGAGTAACAGTTCC	Verify T-DNA
NTLB5-LB	AATCCAGATCCCCCGAATTA		Verify T-DNA
OE-LPL2	GGTACCATGGCCATCCCCGTCGAGG (Kpn I)	ACTAGTTCAAGTAGCTCTCTGTGGCAATG (Spe I)	Ubi:LPL2
35S-LPL2	TCTAGAATGGCCATCCCCGTCGAGG (Xba I)	GGTACCTCAAGTAGCTCTCTGTGGCAATG (Kpn I)	35S:LPL2
LPL2-Y2H	GAATTCATGGCCATCCCCGTCG (EcoR I)	CGAGCTCTCAAGTAGCTCTCTGTGGC (Sac I)	Y2H (+AD)
LPL3-Y2H	CCCGGGGATGGCCCATGTTTCGTTC (Sma I)	GGGATCCTTATTTGTAGGATAGGGGGCC (BamH I)	Y2H (+BD)
LPL3-FP	GTAACGAGGCTTTGATTGCC	CGCACAAAAGAGCAAAAGAG	Verify T-DNA
LPL3-SP		AGTCGCTAATTTCTTCACCAAGG	Verify T-DNA
PIR-FP	GCTGCATACCGGTTGTCTCTA	CAACAAGAACAGGTAGAACCCG	Verify T-DNA
PIR-SP	TGGTTCACGTAGTGGGCCATCG		Verify T-DNA

Underlined regions introduce different enzyme sites

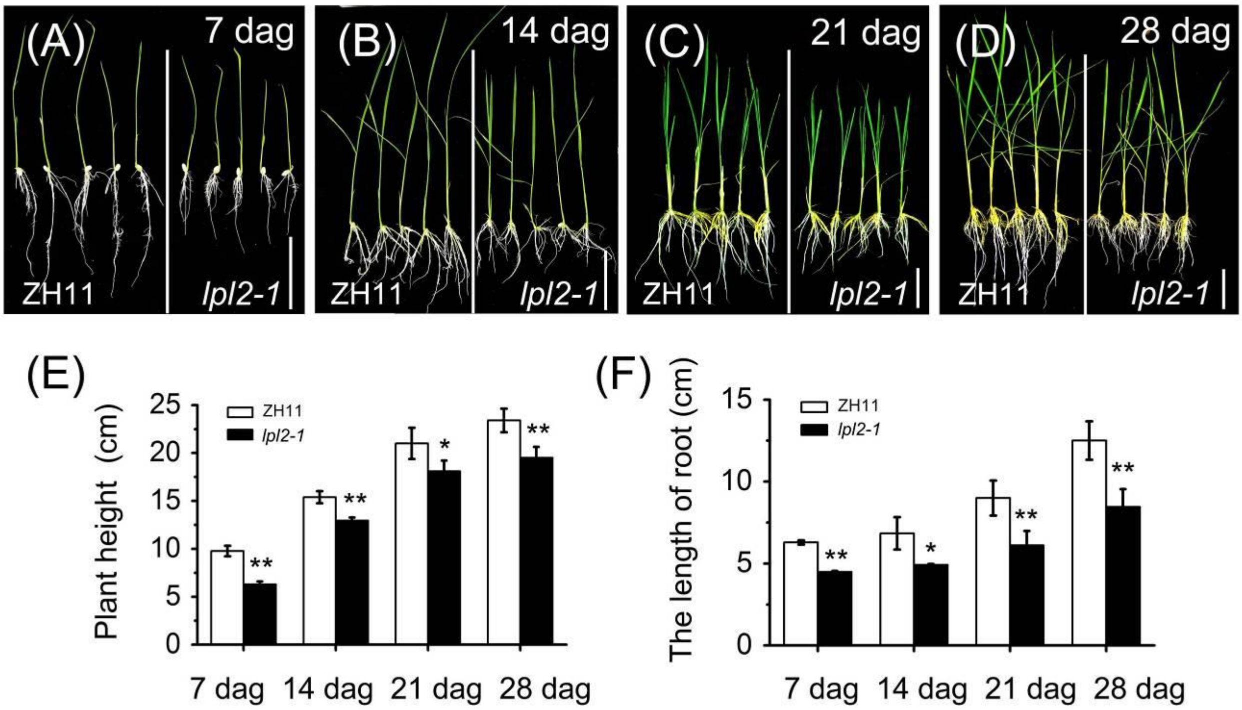

Supplementary Fig. S1. Plant height and root length of ZH11 and *lpl2-1* at the seedling stage. (A–D) Seedling of ZH11 and *lpl2-1* at 7 days after germination (dag), 14 dag, 21 dag and 28 dag. (E) Plant height of ZH11 and *lpl2-1*. (F) The root length of ZH11 and *lpl2-1*. The data presented the mean values (n = 15). The experiment was repeated at least three times. Error bars show SD; *, ** are significant at the 5% and 1% probability level, respectively (Student's *t*-test). Bars = 1 cm.

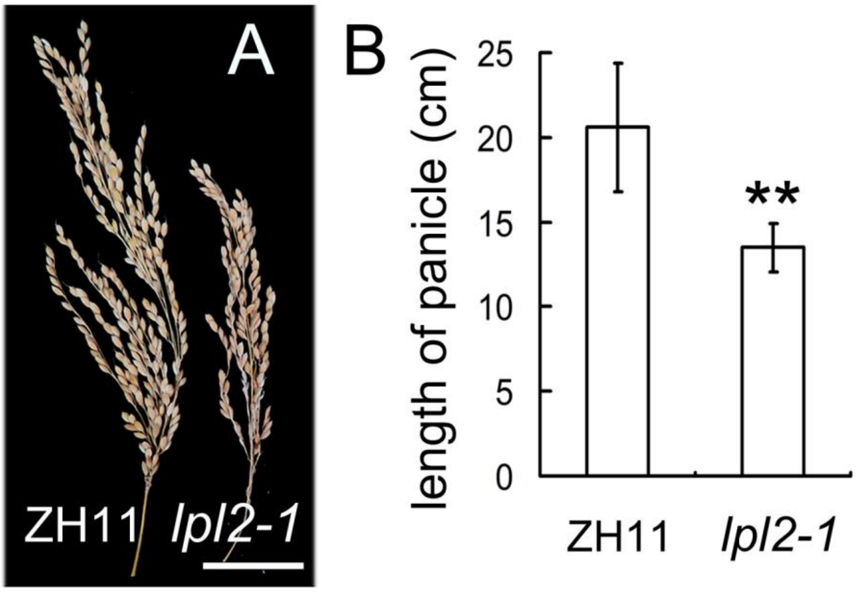

Supplementary Fig. S2. Phenotype of spikes in ZH11 and *lpl2-1*. (A) Spikes of ZH11 and *lpl2-1*. Yield decreased and fertility reduced. (B) Length of panicle in ZH11 and *lpl2-1*. The data presented the mean values (n = 30). Error bars show SD; ** indicates that the means are significantly different at P < 0.01 (Student's *t*-test). Bar = 5 cm.

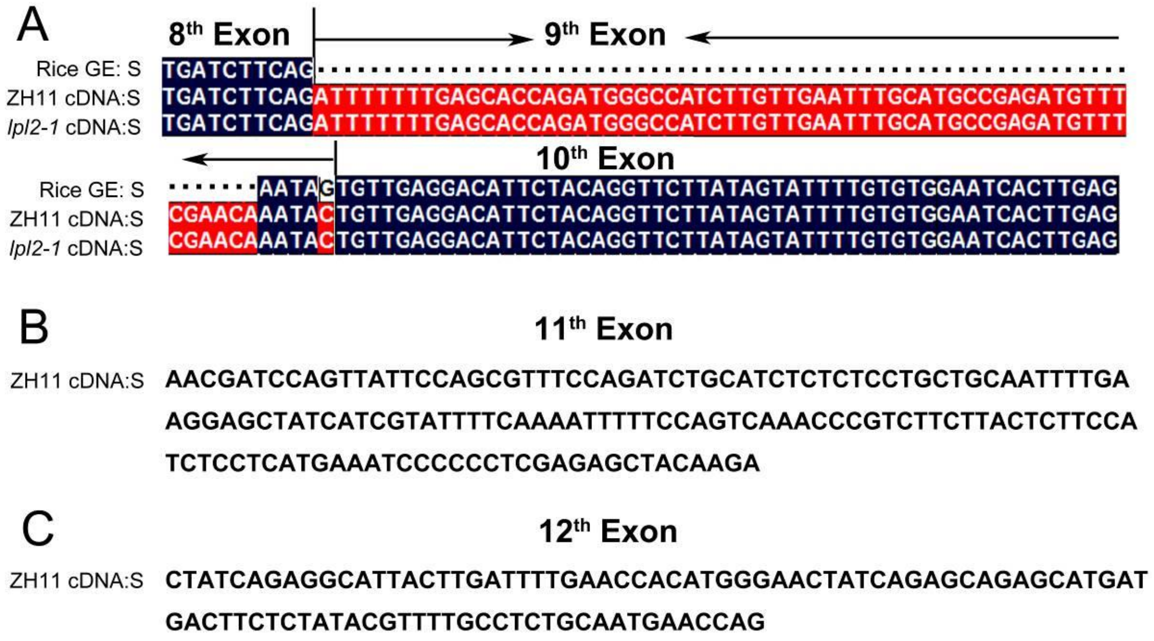

Supplementary Fig. S3. Supplement of *LPL2* cDNA sequence. (A) Supplement of *LPL2* cDNA sequence in 9th exon sequence by adding 65 bp. Rice GE:S represents predicted *LPL2* cDNA sequence from RiceGE; ZH11 cDNA:S shows *LPL2* cDNA sequence from ZH11 cDNA by sequencing; *lpl2-1* cDNA:S represents *LPL2* cDNA sequence from *lpl2-1* by sequencing. (B, C) Supplement of *LPL2* cDNA sequence in 11th exon (adding 152 bp) and 12th exon (adding 94 bp) from ZH11 cDNA.

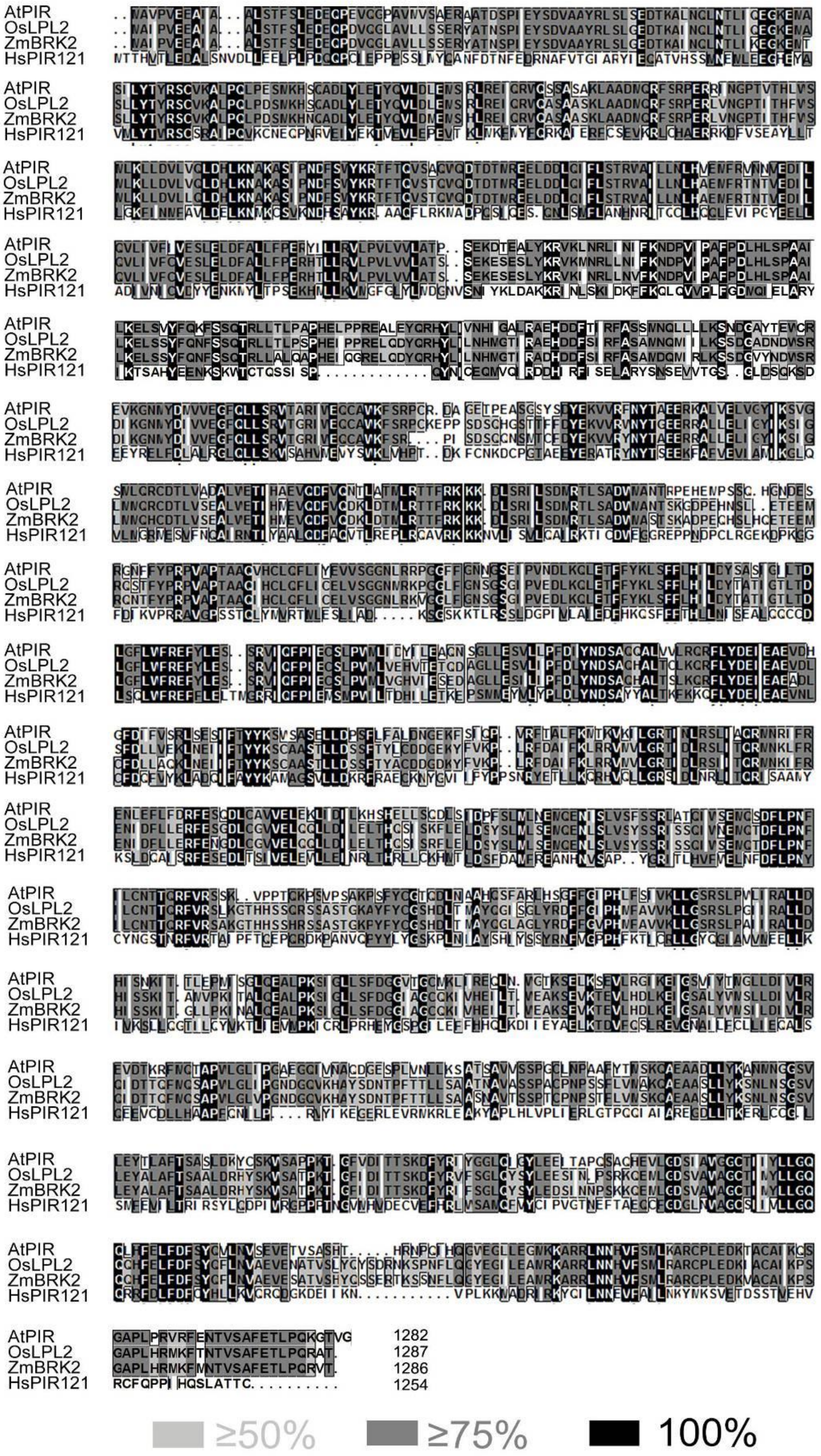

Supplementary Fig. S4. Amino acid alignment of Arabidopsis PIR, rice LPL2, maize BRK2 and human PIR121.

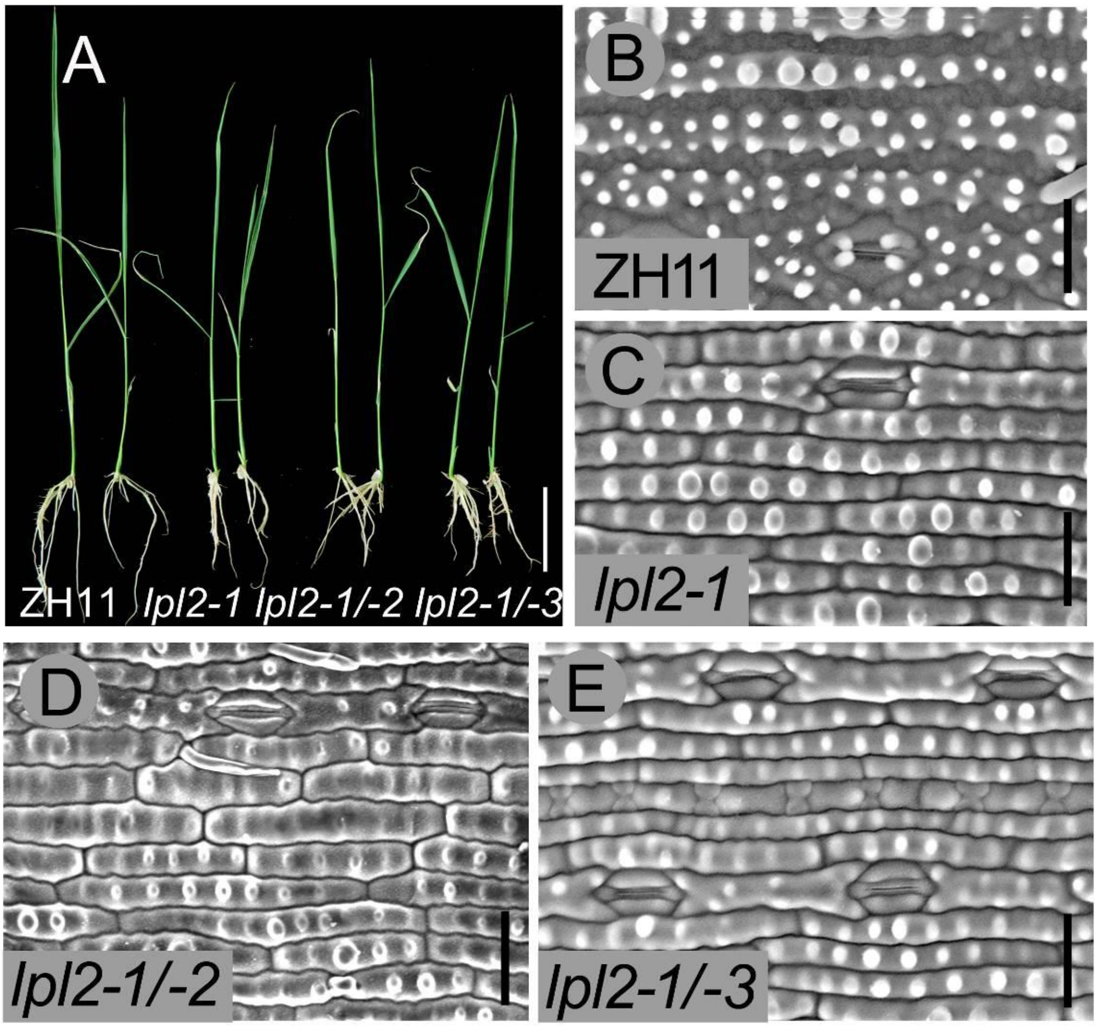

Supplementary Fig. S5. Seedlings and leaf abaxial epidermis of ZH11, *lpl2-1*, *lpl2-1/-2* and *lpl2-1/-3*. (A) Two-week-old seedlings of ZH11, *lpl2-1*, *lpl2-1/-2* and *lpl2-1/-3*. Bar = 2 cm. (B–E) SEM images of abaxial leaf surfaces of ZH11 (B), *lpl2-1* (C), *lpl2-1/-2* (D) and *lpl2-1/-3* (E). Bars = 20 μm.

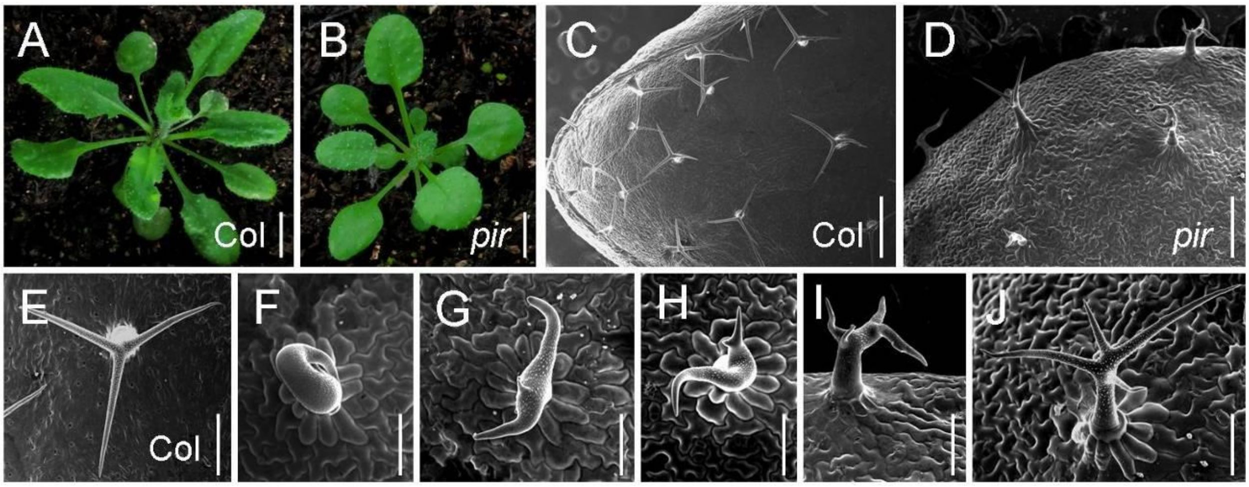

Supplementary Fig. S6. Plant and trichomes of adaxial leaves in Col and *pir*. (A, B) Two-week-old seedlings of wild type (A) and *pir* (B). Bars = 1 cm. (C, E) leaf trichomes of Col. (D, F–J). *pir* leaf trichomes. Bars = 200 µm.

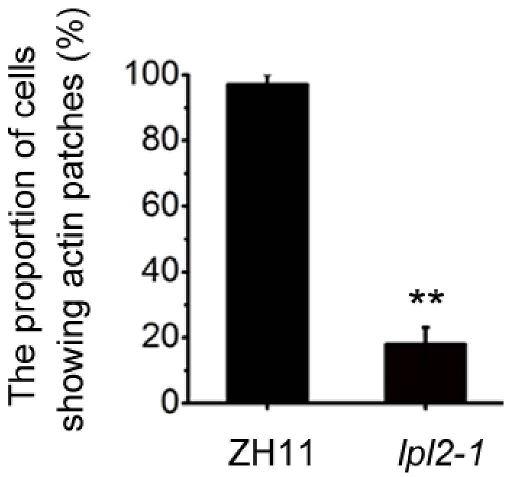

Supplementary Fig. S7. The proportion of cells showing actin patches in ZH11 (Fig. 7C) and *lpl2-1* (Fig. 7G). The proportion (%) = (Number of cells showing actin patches / Number of cell observed) × 100. Error bars show SD, (n = 150); ** indicates that the means are significantly different at $P < 0.01$ (Student's *t*-test).

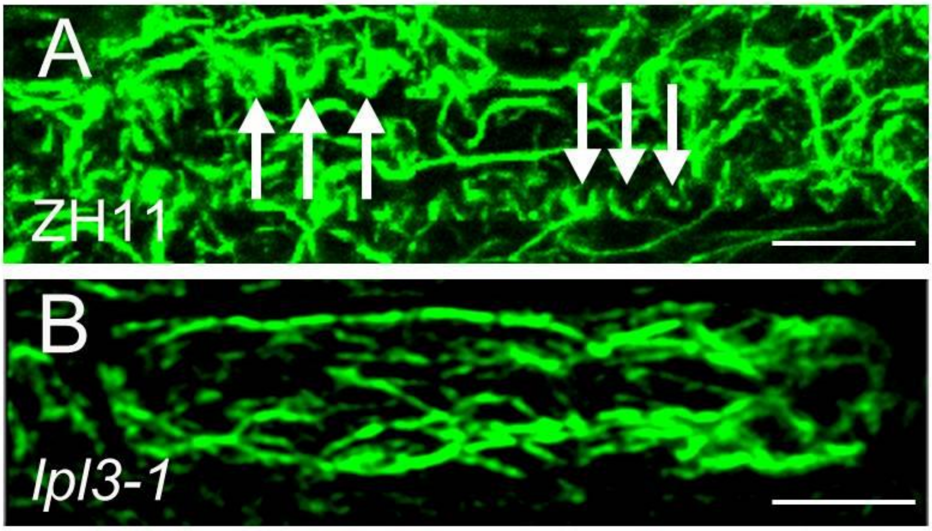

Supplementary Fig. S8. Analysis of actin cytoskeletal structures in ZH11 and *lpl3-1* mature leaf epidermal cells. (A) ZH11. (B) *lpl3-1*. Arrows indicate some of the cortical F-actin enrichments associated with lobe formation. Three independent plants were analyzed. Bars = 10 μm.

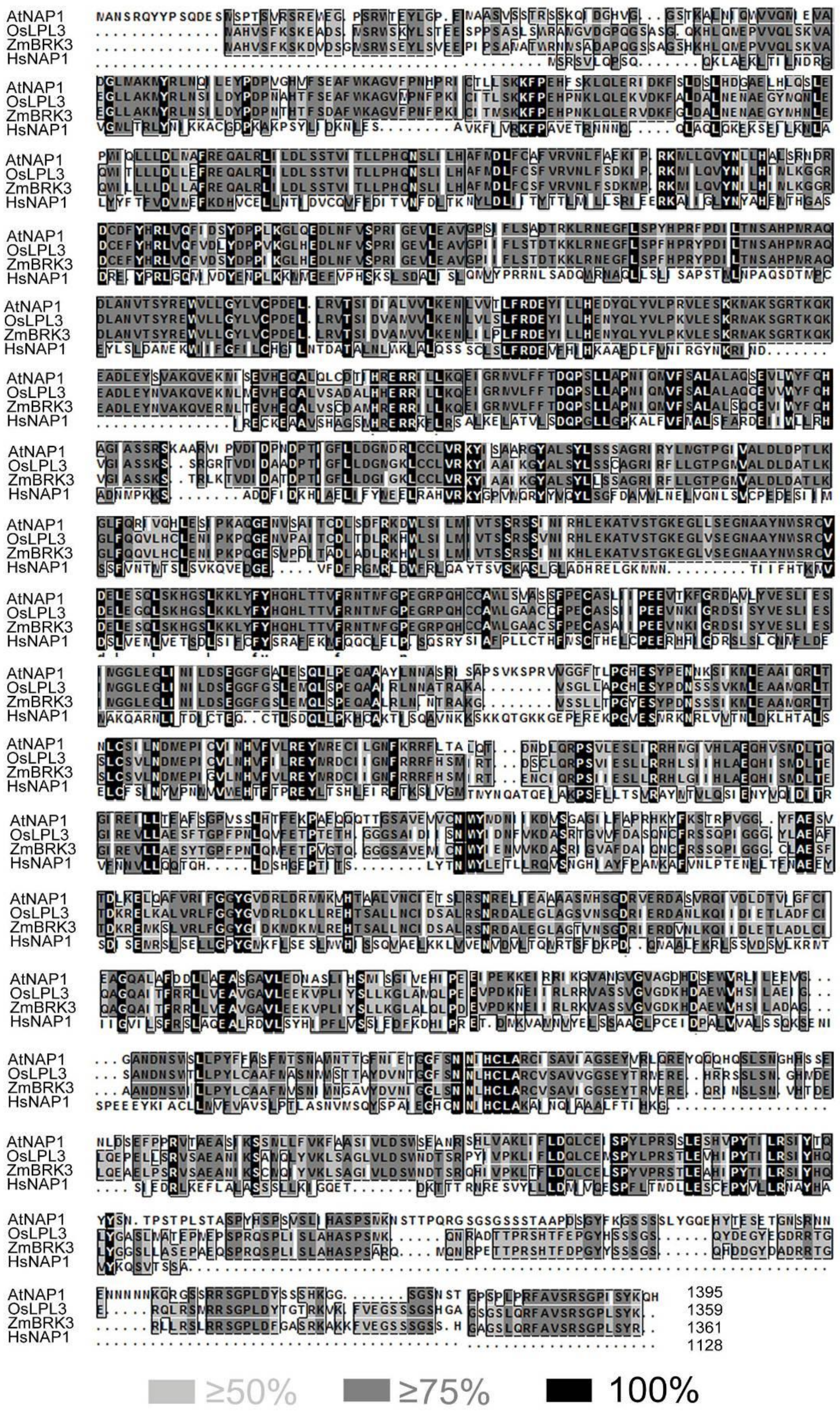

Supplementary Fig. S9. Amino acid alignment of Arabidopsis NAP1, rice LPL3, maize BRK3 and human NAP1.
